# Leptin-mediated meta-inflammation may provide survival benefit in patients receiving maintenance immunotherapy for extensive-stage small cell lung cancer (ES-SCLC)

**DOI:** 10.1007/s00262-023-03533-0

**Published:** 2023-09-05

**Authors:** Emanuele Vita, Alessio Stefani, Geny Piro, Luca Mastrantoni, Marco Cintoni, Giuseppe Cicchetti, Ileana Sparagna, Federico Monaca, Guido Horn, Jacopo Russo, Diletta Barone, Mariantonietta Di Salvatore, Rocco Trisolini, Filippo Lococo, Ciro Mazzarella, Alessandra Cancellieri, Carmine Carbone, Anna Rita Larici, Maria Cristina Mele, Sara Pilotto, Michele Milella, Giampaolo Tortora, Emilio Bria

**Affiliations:** 1 UOSD Oncologia Toraco-Polmonare, Comprehensive Cancer Center, Fondazione Policlinico Universitario Agostino Gemelli IRCCS, Rome, Italy; 2 UOC Oncologia Medica, Comprehensive Cancer Center, Fondazione Policlinico Universitario Agostino Gemelli IRCCS, Rome, Italy; 3 Università Cattolica del Sacro Cuore, Rome, Italy; 4 UOC Nutrizione Clinica, Fondazione Policlinico Universitario Agostino Gemelli IRCCS, Rome, Italy; 5 UOC Radiologia Toracica e Cardiovascolare, Advanced Radiodiagnostics Center, Fondazione Policlinico Universitario Agostino Gemelli IRCCS, Rome, Italy; 6 UOC Pneumologia Interventistica, Fondazione Policlinico Universitario Agostino Gemelli IRCCS, Rome, Italy; 7 UOC Chirurgia Toracica, Fondazione Policlinico Universitario Agostino Gemelli IRCCS, Rome, Italy; 8 UOC Radioterapia Oncologica, Fondazione Policlinico Universitario Agostino Gemelli IRCCS, Rome, Italy; 9 UOC Anatomia Patologica, Fondazione Policlinico Universitario Agostino Gemelli IRCCS, Rome, Italy; 10 UOC Oncologia Medica, Verona University Hospital Trust, Verona, Italy

**Keywords:** SCLC, Immunotherapy, Metabolomics, Leptin, Adipokines, Obesity

## Abstract

**Background:**

Only few ES-SCLC patients experience long-term survival benefit by maintenance IT. Adipokines-induced metabolic meta-inflammation has been related to enhanced responsiveness to IT in obese patients; however, their prognostic role in SCLC is currently controversial.

**Methods:**

Pre-treatment CT scan was used for determining distribution of abdominal adiposity, and blood samples were collected at fasting for measuring glycemia, insulin, ghrelin, leptin and adipokines (TNF-α, IFN-γ, IL-6 and MCP-1). Patients with known history of DM type II or metabolic syndrome with HOMA index > 2.5 were considered insulin resistant (IR).

**Results:**

In ES-SCLC pts receiving maintenance IT, increased leptin concentration and higher leptin/visceral adipose tissue (VAT) ratio were significantly associated with prolonged PFS. By applying a hierarchical clustering algorithm, we identified a cluster of patients characterized by higher leptin values and lower pro-inflammatory cytokines (TNF-α, IFN-γ and IL-6) who experienced longer PFS (13.2 vs 8.05 months; HR: 0.42 [0.18–0.93] *p* = 0.02) and OS (18.04 vs 12.09 mo; HR: 0.53 [0.25–1.29] *p* = 0.07).

**Conclusions:**

Adipokines can play a crucial role to determining effectiveness of anti-cancer immunotherapy. The role of metabolic immune dysfunctions needs further pre-clinical validation and is currently investigated in the larger prospective cohort.

**Supplementary Information:**

The online version contains supplementary material available at 10.1007/s00262-023-03533-0.

## Background

Small cell lung cancer (SCLC) represents about 15% of all lung cancers and is marked by an exceptionally high proliferative rate, early metastasis and poor prognosis with an expected median overall survival < 12 months. At the time of diagnosis, the majority of patients have metastatic or bulky thoracic disease (extensive stage, ES-SCLC) and are not eligible to potentially curative multimodality therapy [1–3]. Based on the results of two prospective trials (Impower133 and CASPIAN), immune checkpoint inhibitors (ICIs) have been integrated into upfront platinum-based chemotherapy (Cisplatin/Carboplatin + Etoposide), finally changing the treatment paradigm after more than three decades [4–8]. However, only a small subset of patients derives prolonged survival benefit (OS > 18 months), despite SCLC is hypothetically a highly immunogenic disease, due to the high TMB and the frequent association with paraneoplastic syndromes [8–11]. These findings raise the need for a deeper understanding of interactions within cellular and molecular networks of the immune system in ES-SCLC patients.

In recent years, several retrospective studies have correlated obesity to better outcomes in different cancer types treated with ICIs (the so-called “obesity paradox”) [12, 13]. Mechanisms of this effect are unclear, although obesity may alter key inflammatory cytokines and promotes a chronic low-grade inflammation (“meta-inflammation,” MI) that modifies tumor-infiltrating lymphocytes and tumor-associated macrophage populations [14–18]. To date, no reports have been published about the role of obesity-related immune dysfunctions in ES-SCLC treated with chemo-immunotherapy (CHT-IO). To further dissect this question, we conducted a retrospective metabolomics profiling in an exploratory cohort of ES-SCL treated with upfront CHT-IO.

## Materials and methods

### Study population and study design

The PICASSO study is a single-institution observational ambispective study, aimed to perform a multi-scale data analysis to gather novel insights about the immune response in SCLC patients treated with platinum-based chemotherapy and immunotherapy (IO). Here, we present the clinical outcomes analysis according to metabolic baseline assessment of the retrospective exploratory cohort. From November 2019 to March 2022, all ES-SCLC patients (PS ECOG 0–2) treated with first-line CHT-IO (i.e., Cisplatin/Carboplatin, etoposide and anti-PD1/PD-L1 inhibitors) at Policlinico A. Gemelli (Rome, Italy) were included in the study. Medical records were reviewed in order to obtain information about demography, treatment, outcome and safety. Patients with known history of diabetes mellitus type II (DM II) or metabolic syndrome with HOMA1 index > 2.5 were classified as insulin resistant (IR) [19]. The HOMA1-IR index was calculed according to equation: HOMA1-IR = (FPI × FPG)/22.5, where FPI is fasting plasma insulin concentration (mU/l), and FPG is fasting plasma glucose (mmol/l). Despite such limitations [20, 21], in clinical practice and cohort/epidemiological studies, calculation of HOMA-IR index by a single fasting sample is widely considered an acceptable compromise for assessing IR status.

Radiologic baseline assessment included the following exams: complete chest and abdomen CT scan with contrast, brain imagining (RMI or high-quality CT scan with contrast) and PET-CT scan for patient without extra-thoracic disease. Radiological evaluations were performed with a frequency ranging from 12 to 16 weeks, according to the monitoring requirements for high-cost drugs of the respective national drug regulatory agencies. Radiological tumor response was evaluated according to iRECIST v.1.1 criteria [22]; however, treatment beyond disease progression was allowed according to the clinicians’ judgment.

### Anthropometric measurements

Weight and height were obtained from patient medical records; BMI was calculated using the formula of weight/height2 (kilograms per square meter) and categorized according to the WHO categories: underweight, BMI < 18.5; normal weight, 18.5 ≤ BMI ≤ 24.9; overweight, 25 ≤ BMI ≤ 29.9 and obese, BMI ≥ 30 [23]. Pre-treatment CT scans with contrast, performed according to clinical practice, have been used to assess body composition without additional costs or radiation exposure (slice thickness, 2–5 mm). Measurements were performed at the mid-portion of the L4 vertebral body using an in-house software (SliceOmatic 5.0 ®) [24] and included cross-sectional areas (CSA) in cm2 of abdominal subcutaneous adipose tissue (SAT), visceral adipose tissue (VAT), intermuscular adipose tissue (IMAT) and total abdominal fat area (TFA). In addition, body composition measurements were standardized by subtracting the mean of each measurement and dividing by the standard deviation (SD).

### Sample collection and processing

Pre-treatment blood samples were collected at fasting for measuring glycemia, insulin, C-peptide, ghrelin and adipokines (TNF-α, IFN-γ, IL-6, leptin and MCP-1). Metabolic analytics detection in plasma samples was performed with Luminex XMAP technology (Luminex Performance Human Metabolic Magnetic Panel), according to the manufacturer’s instructions. Data acquisition and analysis were performed by using Bio-Plex Manager software (Bio-Rad®).

### Study objective and statistical analyses

The primary aim of this analysis is to assess if obesity-related immune dysfunction can affect clinical outcomes in ES-SCLC patients receiving maintenance immunotherapy. The measured clinical outcomes were median progression-free survival (PFS) and median overall survival (OS). PFS was defined as the time from treatment initiation to disease progression or death, whichever occurred first. OS was defined as the time from treatment initiation to death. For PFS as well as for OS, patients without events were considered as censored at the time of the last follow-up. Median period of follow-up was calculated according to the reverse Kaplan–Meier method.

Baseline patient characteristics were reported with descriptive statistics. Median PFS and median OS were evaluated using the Kaplan–Meier method. In order to identify potentially clinical and metabolomics prognostic factors, we conducted univariate and multivariate Cox regression analyses to estimate hazard ratios (HR) with 95% CIs. The alpha level for all analyses was set to *p* < 0.05. The key clinical covariates were: age (< 70 vs ≥ 70 years old), gender (male vs female), Eastern Cooperative Oncology Group-Performance Status (ECOG-PS) (0 vs 1–2), disease extension (thoracic “bulky” disease vs extra-thoracic metastases), liver metastasis, baseline LDH values (normal vs upper normal limit), BMI cutoff (BMI < 25: underweight/normal vs BMI ≥ 25: overweight/obesity) and onset of insulin resistance (yes vs not).

Spearman correlation coefficient was used to test for correlation between continuous variables. Based on preliminary findings, a multivariate logistic model was designed, and the discriminative power was assessed by calculating the area under the receiver operating characteristic (ROC) curves (AUC) to identify the best cutoff. According to the Levene test, appropriate *t*-tests (Student or Welch) were applied for comparing mean concentration of metabolic analytics in plasma between subgroups. An exploratory hierarchical cluster analysis was performed to evaluate potentially prognostic clusters according to cytokine expression. Lower values were considered equal to the sensibility limit.

## Results

### Patients’ characteristics

From November 2019 to March 2022, 52 consecutive ES-SCLC pts were included. After a median follow-up of at 19.42 months (95% CI 14.38–25.67), 37 patients had completed CHT-IO induction and received maintenance IO. Baseline characteristics are summarized in Table 1. The vast majority of patients were current or former smoker (97%). At baseline evaluation, 57% of patients had extensive intrathoracic diseases defined by VALG criteria (T3–4 due to multiple lung nodules that are too extensive or have tumor/nodal volume that is too large to be encompassed in a tolerable radiation plan), while 43% of patients had distant metastases; no patient had brain metastases. The majority of patients were treated with carboplatin-containing (84%) chemotherapy, associated with atezolizumab (64.9%) or durvalumab (29.7%). Fifteen patients (40.5%) received post-induction consolidative thoracic radiotherapy and eleven patients (29.7%) received prophylactic cranial irradiation. According to BMI, 49% of the patients were overweight or obese (29.7 and 18.9%, respectively). Eleven patients (56.7%) had a known history of diabetes on treatment with oral antidiabetic medications: Nine patients were receiving metformin alone, and two patients were receiving metformin plus glicosurycs (one patient) and GLP-1 analog (one patient); none of the patients included was on replacement treatment with insulin. Ten patients with normal glycemia at fasting showed increased insulin secrection with HOMA index > 2.5.

**Table 1:** Patients’ characteristics (CHT-IO responders)

CHT-IO responders, *n* = 37 (%)			
*Age* (median, range)	64 yo., 40–81	*Gender*	
< 70 yo ≥ 70 yo	26 (70.3) 11 (29.7)	Male Female	20 (54.1) 17 (45.9)
*ECOG-PS*		*Baseline LDH*	
0 1–2	17 (45.9) 20 (54.1)	Lower than UNL(≤ 250 UI/L) Higher than UNL (> 250 UI/L)	22 (56.8) 15 (43.2)
*Disease extension*		*Liver metastasis*	
Thoracic “bulky” disease Metastatic disease	21 (56.8) 16 (43.2)	NO YES	26 (70.3) 11 (29.7)
*BMI*		*Insulin-resistance status*	
BMI ≥ 25 BMI < 25	18 (48.6) 19 (51.4)	DM2/HOMA index > 2.5 Normal glycemic indices	21 (56.8) 16 (43.2)
*Consolidation thoracic RT*		*PCI*	
Yes No	15 (40.5) 22 (59.5)	No Yes	26 (70.3) 11 (29.7)
*Smoking status*		*Treatment regimens*	
Current Former Never	20 (54.1) 16 (43.2) 1 (2.7)	Cisplatin/Carboplatin Atezo/Durva/Pembro	6 (16.2)/31 (83.8) 24 (64.9)/11 (29.7)/2 (5.4)

*UNL* upper normal limit; *RT* radiotherapy; *PCI* prophylactic cranial radiotherapy, *Atezo* atezolizumab; *Durva* durvalumab and *Pembro* pembrolizumab

### Univariate analysis for clinical variables and anthropometric measurements

At the time of data cutoff, a total of 26 patients (70.27%) had disease progression and twenty patients (54.05%) died. The overall median PFS was 10.5 months (95% CI 8.02–13.21), and the median OS was 16.85 months (95% CI 11.14–23.59). In the univariate analysis*,* absence of liver metastesis was strongly associated with both longer PFS and OS (*p* < 0.0001); overweight and obese patients (BMI ≥ 25) showed also significant longer overall survival compared to patients with BMI < 25 (NR vs 11.37 mo., HR (95%CI): 0.34 (0.14–0.83) *p* = 0.02) (Table S1 and Fig. S1). Next, we analyzed by univariate Cox regression test the potential association beetween adipose dissue (AT) distribution and survival outcomes; in this case, we did not find any statistical significance with PFS and OS, either considering VAT (*p* = 0.57 for PFS; *p* = 0.38 for OS), SAT (*p* = 0.44 for PFS; *p* = 0.36 for OS), IMAT (*p* = 0.29 for PFS; *p* = 0.65) or TFA (*p* = 0.56 for PFS; *p* = 0.65 for OS).

### Univariate analysis for insulin-resistance status

**Fig. 1:**
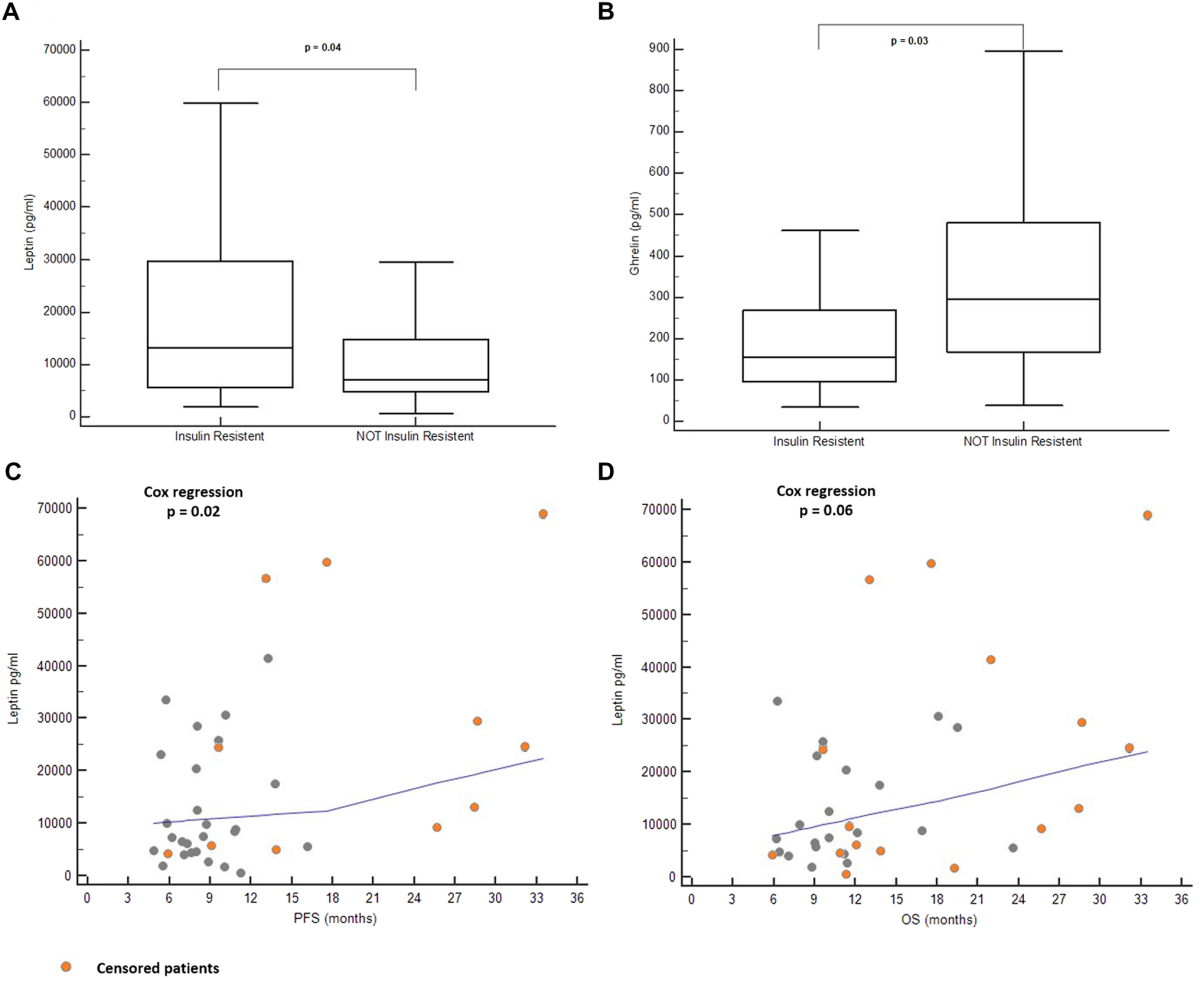
Leptin and ghrelin plasma concentration according to insulin-resistance status. **a** Leptin median concentration comparison between insulin-resistant and not insulin-resistant patients. **b** Ghrelin median concentration comparison between insulin-resistant and not insulin-resistant patients. **c**–**d** Scatter plot showing relationship between leptin values and survival outcome

As previously discussed, the development of insulin resistance is widely considered a clinical epiphenomenon of AT-related meta-inflammation and immune dysfunction. In order to deeper define metabolic profile of out cohort, we investigated if the onset of insulin resistance could be differentially associated with endocrine activity of adipose tissue. Medium leptin plasma concentration was significantly higher in IR patients, compared to NIR patients (*p* = 0.04); conversely, median ghrelin concentration was significantly lower in IR patients compared to NIR (*p* = 0.03) (Fig. 1a–b). In Cox regression analysis, increase leptin values were significantly associated with either prolonged PFS (*p* = 0.02) and OS (*p* = 0.06) (Fig. 1c–d); conversely, no association was found between ghrelin values and survival outcomes (*p* = 0.69 for both PFS and OS). Considering adipose tissue distribution, increased VAT area was significantly associated with insulin resistance (Spearman rho = 0.36; *p* = 0.02); conversely, this association was weaker with SAT area (Spearman rho = 0.30; *p* = 0.06) and non-significant with IMAT (Spearman rho = 0.05; *p* = 0.76).

### Selection of a multivariate metabolic model for PFS and OS

**Fig. 2:**
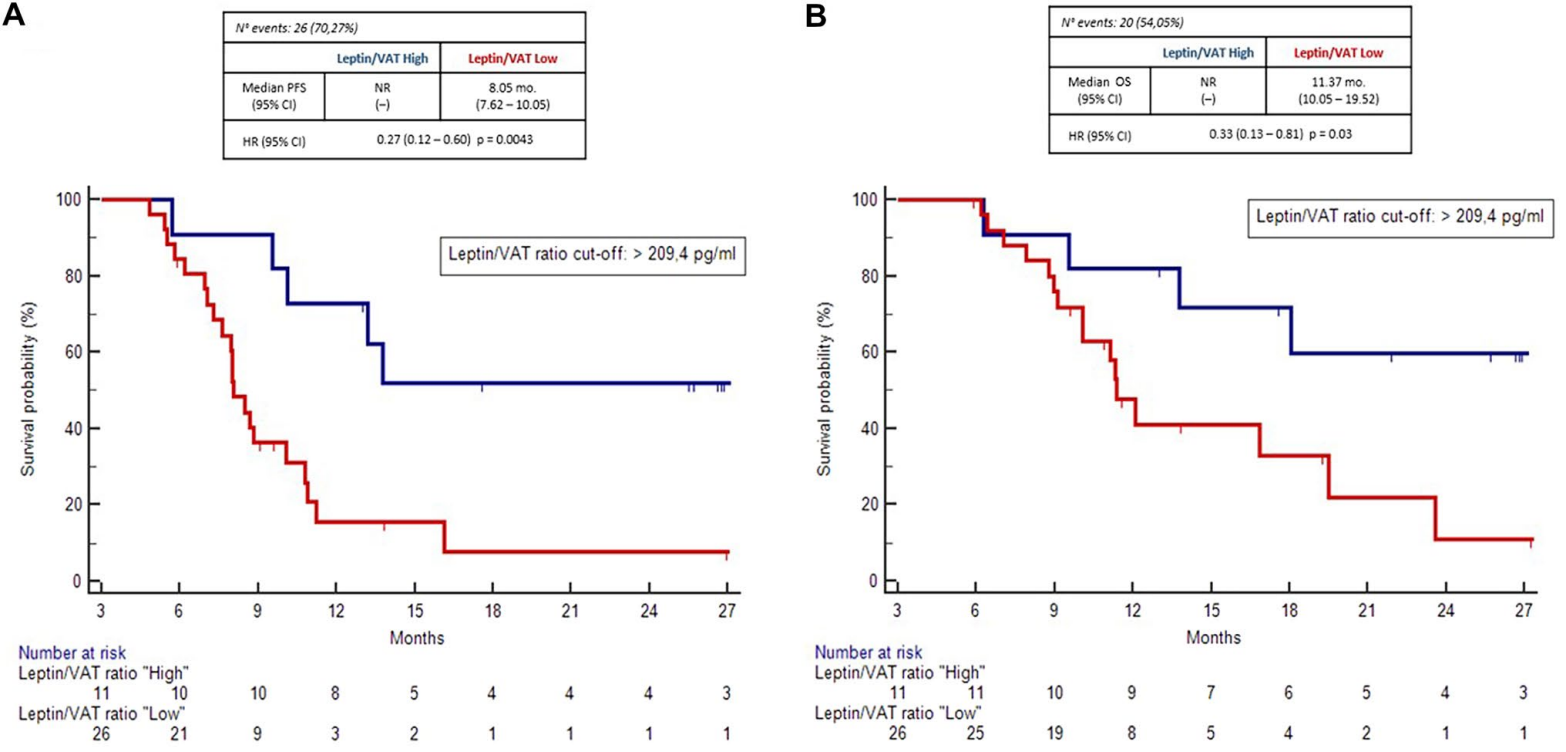
Kaplan–Meier curves for PFS (**a**) and OS (**b**) according to leptin/VAT ratio

With the purpose to quantify adipokine secretion activity in each patient and its correlation with the development of the meta-inflammation seen in obesity, we measured the ratio beetween absolute blood leptin values and visceral adiposity area (leptin/VAT ratio). By ROC curve analysis, we established a discriminative cutoff value for the onset of insulin resistance (leptin/VAT ratio > 209.4 pg/cm^2^). At univariate analysis, patients with higher leptin/VAT ratio showed significant longer PFS compared to patients with lower leptin/VAT ratio (NR vs 8.05 months [7.62–10.05]; HR: 0.27 [0.12–0.60] *p* = 0.0043). Despite immature survival data (54% of the events), OS was also significative longer in high leptin/VAT subgroup (NR vs 11.37 mo. [10.05–19.52]; HR: 0.33 [0.13–0.81] *p* = 0.03) (Fig. 2).

In the multivariate Cox regression analysis, including significant clinical variables, absence of liver metastases (*p* = 0.0001) and “high” leptin/VAT ratio (*p* = 0.01) confirmed statistical significance as prognostic factor for PFS. Regarding to OS analysis, absence of liver metastases confirmed to be associated with better outcomes (*p* = 0.0002); conversely, “high” leptin/VAT ratio did not reached stastical significance, despite a positive trend toward association was still confirmed (*p* = 0.27), considering the early follow-up evalutation (Table 2).

**Table 2:** Multivariate analysis for PFS and OS

	PFS median (mo.) HR (CI 95%)	OS median (mo.) HR (CI 95%)
Liver metastasis (No vs Yes)	**p = 0.0001**	**p = 0.0002**
Body mass index (BMI ≥ 25 vs BMI < 25)	NA	p = 0.87
Insulin resistance Yes vs No	p = 0.10	NA
Leptin/VAT ratio (High vs low)	**p = 0.01**	*p* = *0.27*
Leptin/adipokines clusters (Cluster A vs Cluster B)	**p = 0.01**	***p* = *0.07***

### Blood cytokine profiling and its relations with survival outcomes

We analyzed plasma concentration of four pro-inflammatory cytokines (TNF-alfa, IFN-gamma, IL-6 and MCP-1) that have reported as dysregulated in AT-associated immune disorders. In patients with “high” leptin/VAT ratio, we found significantly lower median concentrion of IFN-gamma (*p* = 0.02) and TNF-alfa (*p* = 0.03), comperated to subgroup of patients with “low” leptin/VAT ratio. Significant interaction was not reached for median concentration of IL-6, despite a trend toward association was still evidenced (*p* = 0.07). No difference was found beetwen median concentraion of chemokine MCP-1 (*p* = 0.53) (Fig. S2).

**Fig. 3:**
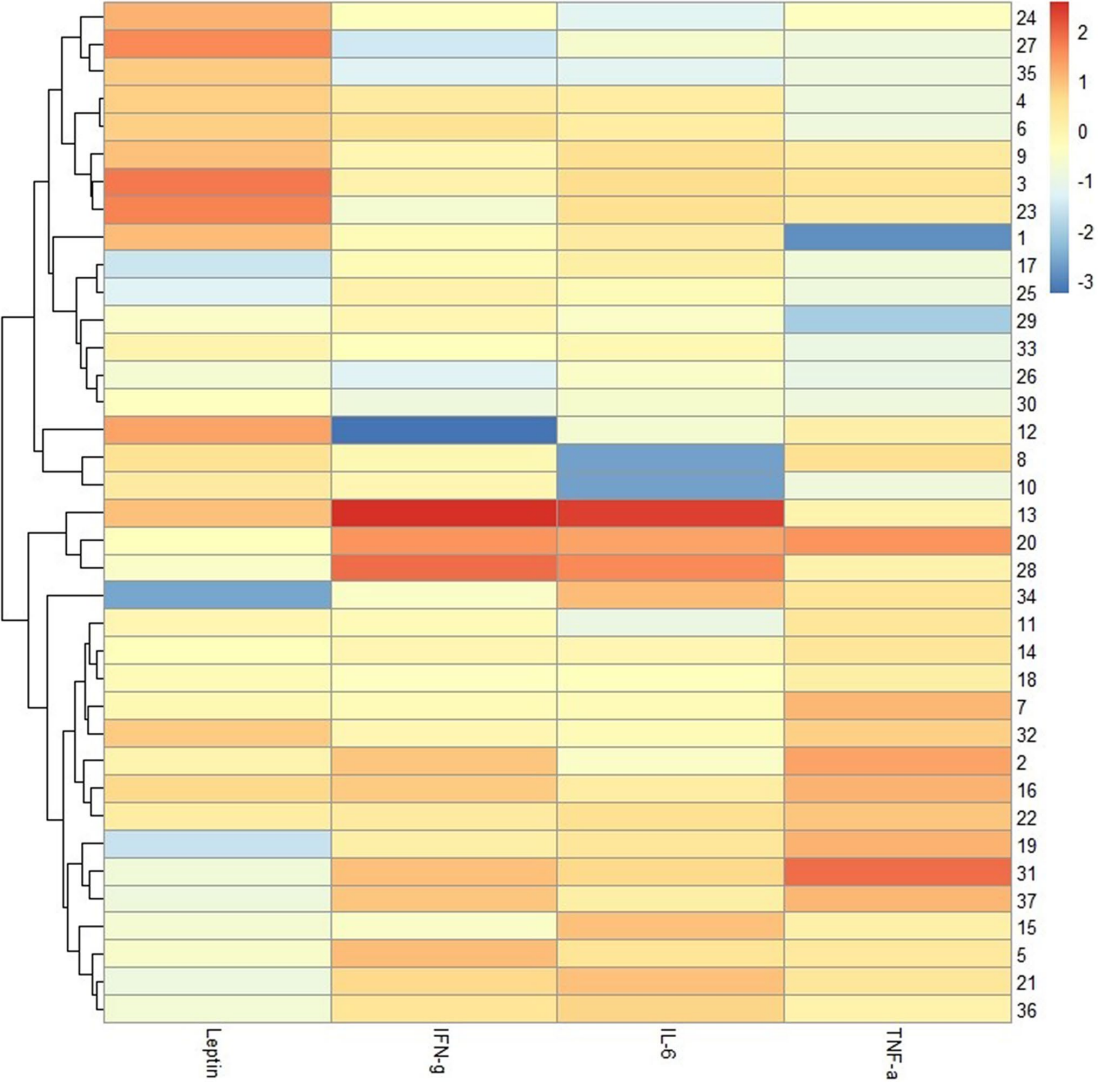
Heatmap evaluating leptin and cytokine expression. Leptin and cytokine values were log-transformed and normalized. A hierarchical clustering algorithm using the complete-linkage method was performed to generate clusters according to leptin, IFN-gamma, IL-6 and TNF-alfa expression. Lower values were considered equal to the sensibility limit

**Fig. 4:**
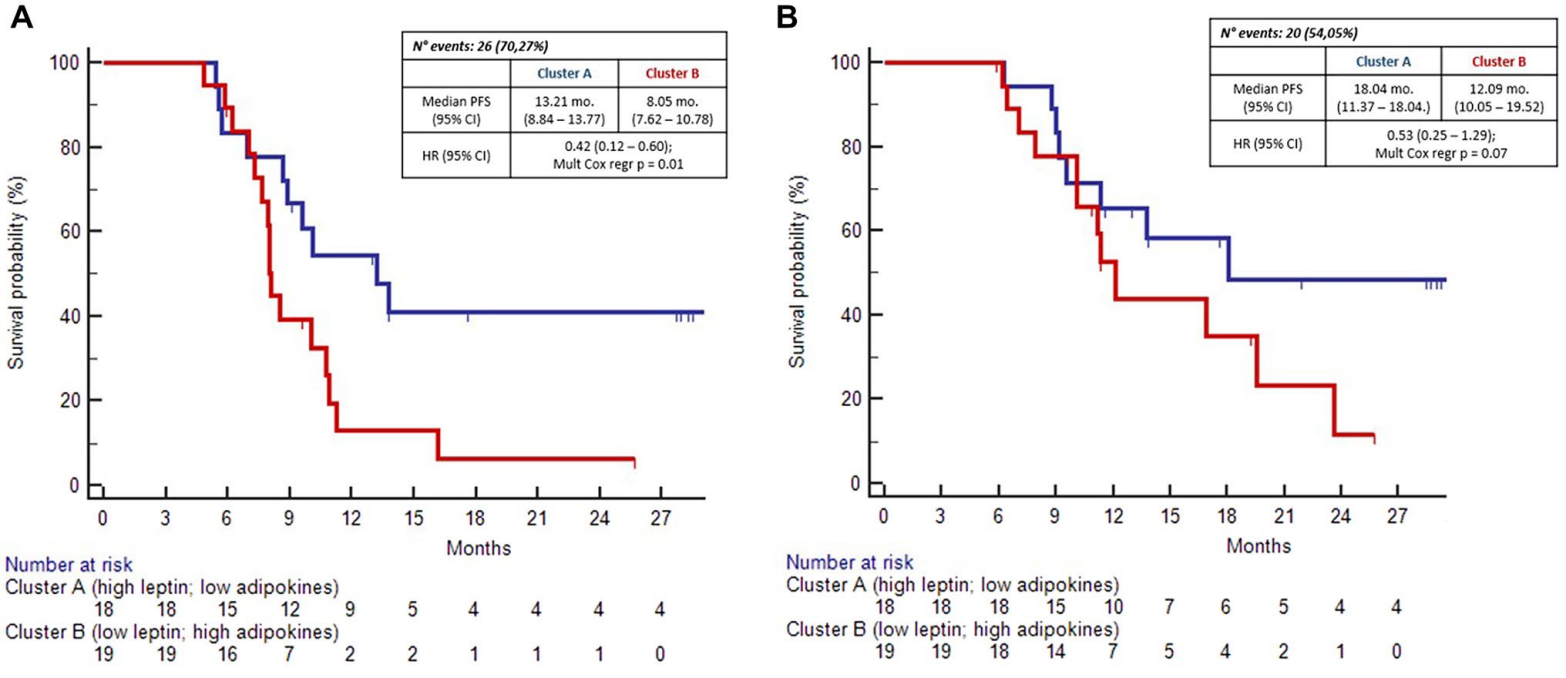
Kaplan–Meier curves for PFS (**a**) and OS (**b**) according to leptin/adipokines clusters

Finally, by applying a hierarchical clustering algorithm, we identified two clusters according to leptin, TNF-alfa, IFN gamma and IL-6 expression (Fig. 3). At multivariate Cox analysis, patients in the cluster with higher leptin and lower inflammotory cytokine expression showed significant longer PFS compared to patients with lower leptin and higher inflammotory cytokine expression (13.2 vs 8.05 months; HR: 0.42 [0.18–0.93] *p* = 0.02). Similary, a strong trend toward significance was observerd for OS (18.04 vs 12.09 mo; HR: 0.53 [0.25–1.29] p = 0.07) (Fig. 4).

## Discussion

Despite the recent introduction of immunotherapy in treatment algorithms, SCLC remains a highly lethal disease, particularly for chemorefractory patients who have very dismal prognosis. Unfortunately, also only few patients who reached IO maintenance phase experience durable survival benefit [8]. Consequently, there is an urgent need for a multilevel integration of immune assays in order to understand how best to implement immunotherapy, who would benefit the most, and finally, how to extend the durable benefit of effective antitumor immunity to a greater fraction of patients. Adipose tissue, as energy storage and major endocrine active organ, produces a variety of bioactive proteins (adipokines) which promote the entirety of metabolism alterations, globally termed as metabolic syndrome. However, even before the overt conditions of the metabolic syndrome, such as insulin-resistance and cardiovascular disease, adipokines secretion contributes to the establishment and maintenance of local and systemic inflammation and consequent suboptimal immune responses in obese individuals [25, 26]. Particularly, Wang et al. showed that in mice models, high levels of leptin increase T-cell aging and upregulation of PD-1, leading to tumorigenic effect; however, the PD1-mediated T-cell dysfunction can be effectively reversed by ICIs treatment [14–16]. Importantly, adipose inflammation and its protumor consequences can be found in some individuals who are not considered obese or overweight according to BMI. Conversely, a subgroup of obese individuals (metabolic healthy obesity—MHO) is free of the cardiometabolic risk factors and maintain normal insulin sensitivity, emphasizing the central role of adipose tissue distribution and function in metabolic health [26, 27].

In our report, anthropometric measurements (BMI and body adiposity distribution) and insulin resistance are suggestive but not conclusively related with survival benefit in ES-SCLC receiving maintenance IO. According to the concept of metabolically unhealthy/healthy obesity, we then investigated the role of adipokines secretion as clinical marker of systemic meta-inflammation. Abdominal obesity makes a greater contribution to opposite profiles of leptin/ghrelin secretion leading to the onset of insulin resistance and abnormal blood pro-inflammatory cytokines values. Consequently, we designed a multivariate regression model (leptin/VAT ratio) by normalizing leptin values in order to deeper quantify the endocrine activity of AT and avoid many factors that can affect anthropometric measures (such as gender, race, high muscle mass and hydration status).

In multivariate Cox analysis, leptin/VAT ratio showed statistically significant association with PFS (*p* = 0.03); in the same analysis for OS, statistical significance was not reached (*p* = 0.27), probably due to immaturity of survival FU (54% of the events); however, a trend toward association was still evidenced. Finally, we applied a hierarchical clustering algorithm and identified a cluster of patients (Cluster A: high leptin/low pro-inflammatory cytokines) who experience prolonged survival benefit by IO maintenance treatment. Cluster A was strongly associated with better PFS (*p* = 0.01) but, again, only a borderline significance was reached for OS (*p* = 0.07), probably due to small sample size. However, in multivariate analysis, both leptin/VAT ratio and leptin/adipokines clusters resulted significant prognostic factors, as well as liver metastasis reported in subgroup analysis of pivotal trials [8].

We acknowledge several limitations to this study. The cohort was small (52 patients), the study is retrospective and prognostic variable is analyzed into a highly selected patients cohort (non-platinum refractory patients). However, into the context of a hypothesis-generator study, we identified two refined and meaningful multivariate metabolic models (leptin/VAT ratio and leptin/adipokines clusters) that are extraordinary consistent with leptin-mediated T-cell dysregulations described both in obese mice [14] and retrospective clinical cohort studies [28–31]. Consequently, in our opinion, the metabolic profiling of SCLC patients deserves further investigations and validation, particularly in relation with newly emerging prognostic/predictive variable (PD-L1 expression on tumor cells and peripheral T cells, genomic/transcriptomic signatures and classifications).

## Conclusions

In our report, we confirmed in a homogeneous population of non-chemorefractory patients, the association between leptin-mediated overt metabolic disorders, blood pro-inflammatory cytokine profile and responsiveness to maintenance IO. The role of metabolic immune dysfunctions needs further pre-clinical validation and is currently investigated in the larger prospective cohort of the PICASSO study.

### Supplementary Information

Below is the link to the electronic supplementary material.

Supplementary file1 (DOCX 215 KB)

## Data Availability

The data presented in this study are available on request from the corresponding author after approval of local ethical committee, according to Italian Data Protection Authority.

## References

[1] Rudin CM, Brambilla E, Faivre-Finn C, Sage J (2021) Small-cell lung cancer. Nat Rev Dis Primers 7(1):3. 10.1038/s41572-020-00235-0. 10.1038/s41572-020-00235-0 PMC8177722 33446664

[2] Micke P, Faldum A, Metz T (2002). Staging small cell lung cancer: Veterans Administration Lung Study Group versus International Association for the Study of Lung Cancer–what limits limited disease?. Lung Cancer.

[3] Farago, AF, Keane FK (2018) Current standards for clinical management of small cell lung cancer. Transl Lung Cancer Res 7:69–79. 10.21037/tlcr.2018.01.16 PMC5835595 29535913

[4] Paz-Ares L, Dvorkin M, Chen Y (2019). Durvalumab plus platinum-etoposide versus platinum-etoposide in first-line treatment of extensive-stage small-cell lung cancer (CASPIAN): a randomised, controlled, open-label, phase 3 trial. Lancet.

[5] Goldman JW, Dvorkin M, Chen Y (2021). Durvalumab, with or without tremelimumab, plus platinum-etoposide versus platinum-etoposide alone in first-line treatment of extensive-stage small-cell lung cancer (CASPIAN): updated results from a randomised, controlled, open-label, phase 3 trial. Lancet Oncol.

[6] Horn L, Mansfield AS, Szczęsna A (2018). First-line atezolizumab plus chemotherapy in extensive-stage small-cell lung cancer. NEJM.

[7] Liu SV, Reck M, Mansfield AS (2021). Updated overall survival and PDL1 subgroup analysis of patients with extensive-stage small-cell lung cancer treated with atezolizumab, carboplatin, and etoposide (IMpower133). J Clin Oncol.

[8] Reck M, Mok TSK, Mansfield A (2022). Brief report: exploratory analysis of maintenance therapy in patients with extensive-stage SCLC treated first line with atezolizumab plus carboplatin and etoposide. J Thorac Oncol.

[9] Bria E, Garassino MC, Del Signore E et al (2022) Atezolizumab (ATZ) plus carboplatin (Cb) and etoposide (eto) in patients with untreated extensive-stage small cell lung cancer (ES-SCLC): Results from the interim analysis of MAURIS trial. ESMO 2022 - Poster session no 1533. Ann Oncol 33(suppl_7):S701–S712. 10.1016/annonc/annonc1074

[10] Garcia Campelo MR, Domine Gomez M, De Castro Carpeno J et al (2022) Primary results from IMfirst, a phase IIIb open label safety study of atezolizumab (ATZ) + carboplatin (CB)/cisplatin (CP) + etoposide (ET) in an interventional real-world (RW) clinical setting of extensive-stage small cell lung cancer (ES-SCLC) in Spain. ESMO 2022 – Poster session no. 1531P. Ann Oncol 33(suppl_7):S701–S712. 10.1016/annonc/annonc1074

[11] Isla D, Arriola E, M.R. Garcia Campelo MR et al (2022) Phase IIIb study of durvalumab plus platinum-etoposide in first-line treatment of extensive-stage small cell lung cancer (CANTABRICO): Preliminary efficacy results. ESMO 2022—Poster session no 1532. Ann Oncol (2022) 33 (suppl_7):S701–S712. 10.1016/annonc/annonc1074

[12] McQuade JL, Daniel CR, Hess KR (2018). Association of body-mass index and outcomes in patients with metastatic melanoma treated with targeted therapy, immunotherapy, or chemotherapy: a retrospective, multicohort analysis. Lancet Oncol.

[13] Cortellini A, Bersanelli M, Buti S (2019). A multicenter study of body mass index in cancer patients treated with anti-PD-1/PD-L1 immune checkpoint inhibitors: when overweight becomes favorable. J Immunother Cancer.

[14] Wang Z, Aguilar EG, Luna JI (2019). Paradoxical effects of obesity on T cell function during tumor progression and PD-1 checkpoint blockade. Nat Med.

[15] Shirakawa K, Yan X, Shinmura K (2016). Obesity accelerates T cell senescence in murine visceral adipose tissue. J Clin Invest.

[16] Dudzinski SO, Bader JE, Beckermann KE (2021). Leptin augments antitumor immunity in obesity by repolarizing tumor-associated macrophages. J Immunol.

[17] Clements VK, Long T, Long R, Figley C, Smith DMC, Ostrand-Rosenberg S (2018). Frontline science: High fat diet and leptin promote tumor progression by inducing myeloid-derived suppressor cells. J Leukoc Biol.

[18] Pingili AK, Chaib M, Sipe LM et al (2021) Immune checkpoint blockade reprograms systemic immune landscape and tumor microenvironment in obesity-associated breast cancer. Cell Rep 35(12):109285. 10.1016/j.celrep.2021.109285. 10.1016/j.celrep.2021.109285 PMC8574993 34161764

[19] Matthews DR, Hosker JP, Rudenski AS, Naylor BA, Treacher DF, Turner RC (1985). Homeostasis model assessment: insulin resistance and beta-cell function from fasting plasma glucose and insulin concentrations in man. Diabetologia.

[20] Wallace TM, Levy JC, Matthews DR (2004). An increase in insulin sensitivity and basal beta-cell function in diabetic subjects treated with pioglitazone in a placebo-controlled randomized study. Diabet Med.

[21] Wallace TM, Levy JC, Matthews DR (2004). Use and abuse of HOMA modeling. Diabetes Care.

[22] Seymour L, Bogaerts J, Perrone A et al (2017) RECIST working group. iRECIST: guidelines for response criteria for use in trials testing immunotherapeutics. Lancet Oncol 18(3):e143–e152. 10.1016/S1470-2045(17)30074-8. Epub 2017 Mar 2. 10.1016/S1470-2045(17)30074-8 PMC5648544 28271869

[23] Obesity: preventing and managing the global epidemic. Report of a WHO consultation. World Health Organ Tech Rep Ser. 2000;894:i-xii, 1–253. 11234459

[24] Irving BA, Weltman JY, Brock DW, Davis CK, Gaesser GA, Weltman A (2007). NIH ImageJ and Slice-O-Matic computed tomography imaging software to quantify soft tissue. Obesity (Silver Spring).

[25] Russo S, Kwiatkowski M, Govorukhina N, Bischoff R, Melgert BN (2021) meta-inflammation and metabolic reprogramming of macrophages in diabetes and obesity: the importance of metabolites. Front Immunol 12:746151. 10.3389/fimmu.2021.746151. 10.3389/fimmu.2021.746151 PMC8602812 34804028

[26] Boutens L, Hooiveld GJ, Dhingra S, Cramer RA, Netea MG, Stienstra R (2018). Unique metabolic activation of adipose tissue macrophages in obesity promotes inflammatory responses. Diabetologia.

[27] Barchetta I, Cimini FA, Ciccarelli G, Baroni MG, Cavallo MG (2019). Sick fat: the good and the bad of old and new circulating markers of adipose tissue inflammation. J Endocrinol Investig.

[28] Stienstra R, Stefan N (2013) Tipping the inflammatory balance: inflammasome activation distinguishes metabolically unhealthy from healthy obesity. Diabetologia. 2013;56:2343–2346. 10.1007/s00125-013-3040-8 23995473

[29] Keegan A, Ricciuti B, Garden P et al (2020) Plasma IL-6 changes correlate to PD-1 inhibitor responses in NSCLC. J Immunother Cancer 8(2):e000678. 10.1136/jitc-2020-000678. 10.1136/jitc-2020-000678 PMC7537334 33020238

[30] Shi Y, Liu X, Du J (2022). Circulating cytokines associated with clinical outcomes in advanced non-small cell lung cancer patients who received chemoimmunotherapy. Thorac Cancer.

[31] Schalper KA, Carleton M, Zhou M (2020). Elevated serum interleukin-8 is associated with enhanced intratumor neutrophils and reduced clinical benefit of immune-checkpoint inhibitors. Nat Med.

