# Supplementary material for: Leptin-mediated meta-inflammation may provide survival benefit in patients receiving maintenance immunotherapy for extensive-stage small cell lung cancer (ES-SCLC)
Source: Cancer Immunol Immunother. 2023 Sep 5;72(11):3803–12. doi: 10.1007/s00262-023-03533-0 (PMC10576666; doi:10.1007/s00262-023-03533-0)
Supplement: Supplementary file 1 — Supplementary file1 (DOCX 215 KB) [file 262_2023_3533_MOESM1_ESM.docx]

Leptin-mediated Meta-inflammation may provide survival benefit in patients receiving maintenance immunotherapy for Extensive-Stage Small Cell Lung cancer (ES-SCLC)

**SUPPPLEMENTARY MATERIALS**

Table S1. Univariate analysis for PFS and OS according to pre-planned clinical variables…………………………………………………………………………………...……………...2

[Fig. S1 Survival outcomes according to BMI. PFS (A) and OS (B) according to BMI. ...2](#_Toc118754796)

Fig. S2 Boxplot of median cytokine blood concentration according to Leptin/VAT ratio subgroup …....3

|  | **PFS median (mo.)**  **HR (CI 95%)** | **OS median (mo.)**  **HR (CI 95%)** |
| --- | --- | --- |
|  |  |  |
| Age  (< 70 yo. vs ≥ 70 yo.) | 8.84 vs 10.05  HR: 1.20 (0.52 – 2.76)  p = 0.65 | 18.04 vs 12.09  HR: 0.81 (0.30 - 2.22)  p = 0.67 |
| Gender  (Male vs Female) | 10.12 vs 8.67  HR: 0.53 (0.24 - 1.15)  p = 0.11 | 16.85 vs 13.77  HR: 0.96 (0.39 - 2.31)  p = 0.92 |
| Performance status  (PS ECOG 0 vs 1-2) | 10.78 vs 8.48  HR: 0.52 (0.24 – 1.13)  p = 0.11 | 18.04 vs 13.77  HR: 0.64 (0.26 – 1.55)  p = 0.32 |
| Disease extension  (Thoracic “bulky” vs metastatic) | 10.05 vs 8.48  HR: 0.97 (0.44 – 2.15)  p = 0.95 | 18.04 vs 12.09  HR: 0.90 (0.36 – 2.32)  p = 0.82 |
| Liver metastasis  (NO vs YES) | 11.23 vs 6.93  HR: 0.18 (0.05 - 0.61)  **p = < 0.0001** | 23.59 vs 8.97  HR: 0.12 (0.03 - 0.44)  **p = < 0.0001** |
| Body mass index  (BMI ≥25 vs BMI <25) | 9.56 vs 10.12  HR: 1.46 (0.68 - 3.16)  p = 0.32 | NR vs 11.37  (0.14 – 0.83)  **p = 0.02** |
| Insulin resistance  (YES vs NOT) | 10.88 vs 7.85  HR: 0.48 (0.21 - 1.09)  p = 0.05 | 19.52 vs 13.30  HR: 0.52 (0.20 - 1.32)  p = 0.13 |
| LDH  (≤ UNL vs > UNL) | 9.56 vs 10.12  HR 1.28 (0.58 – 2.81)  p = 0.53 | 19.52 vs 11.37  HR: 0.75 (0.30 – 1.86)  p = 0.53 |

**Table S1. Univariate analysis for PFS and OS according to pre-planned clinical variables**

**Fig. S1 Survival outcomes according to BMI.** PFS (A) and OS (B) according to BMI.


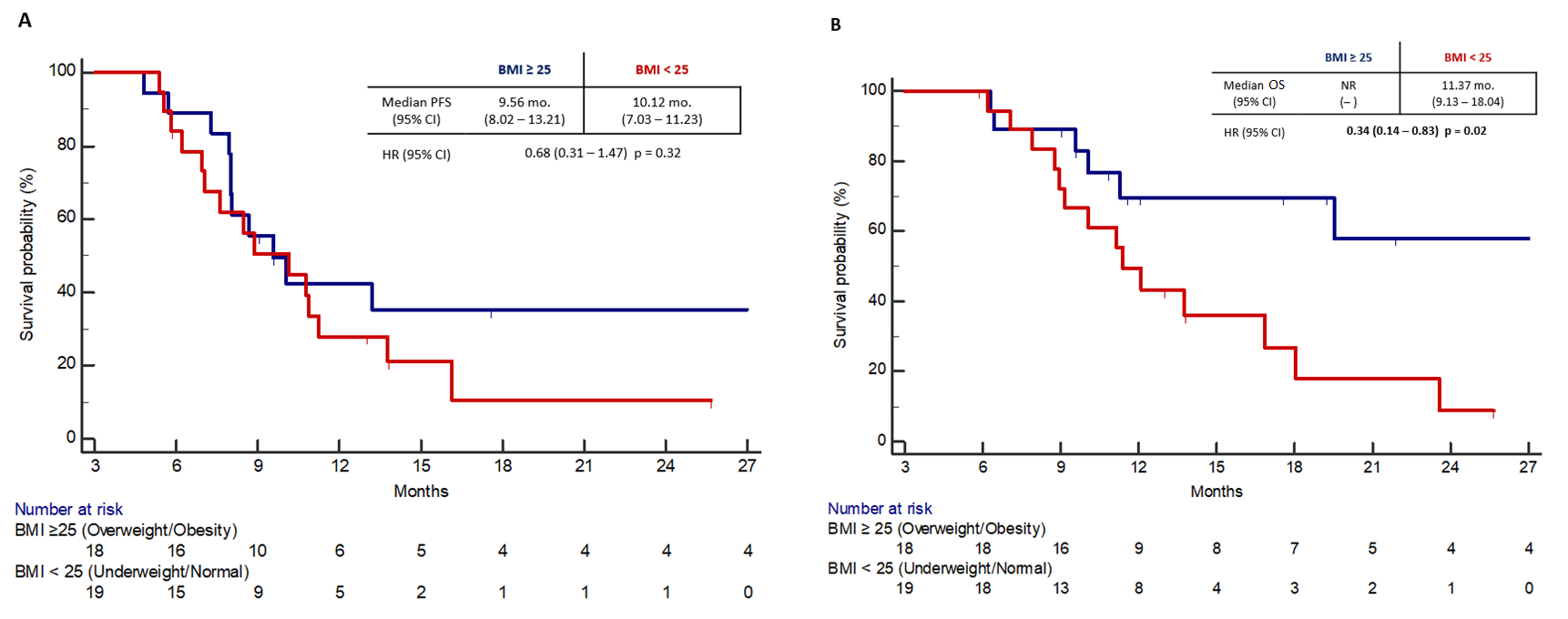


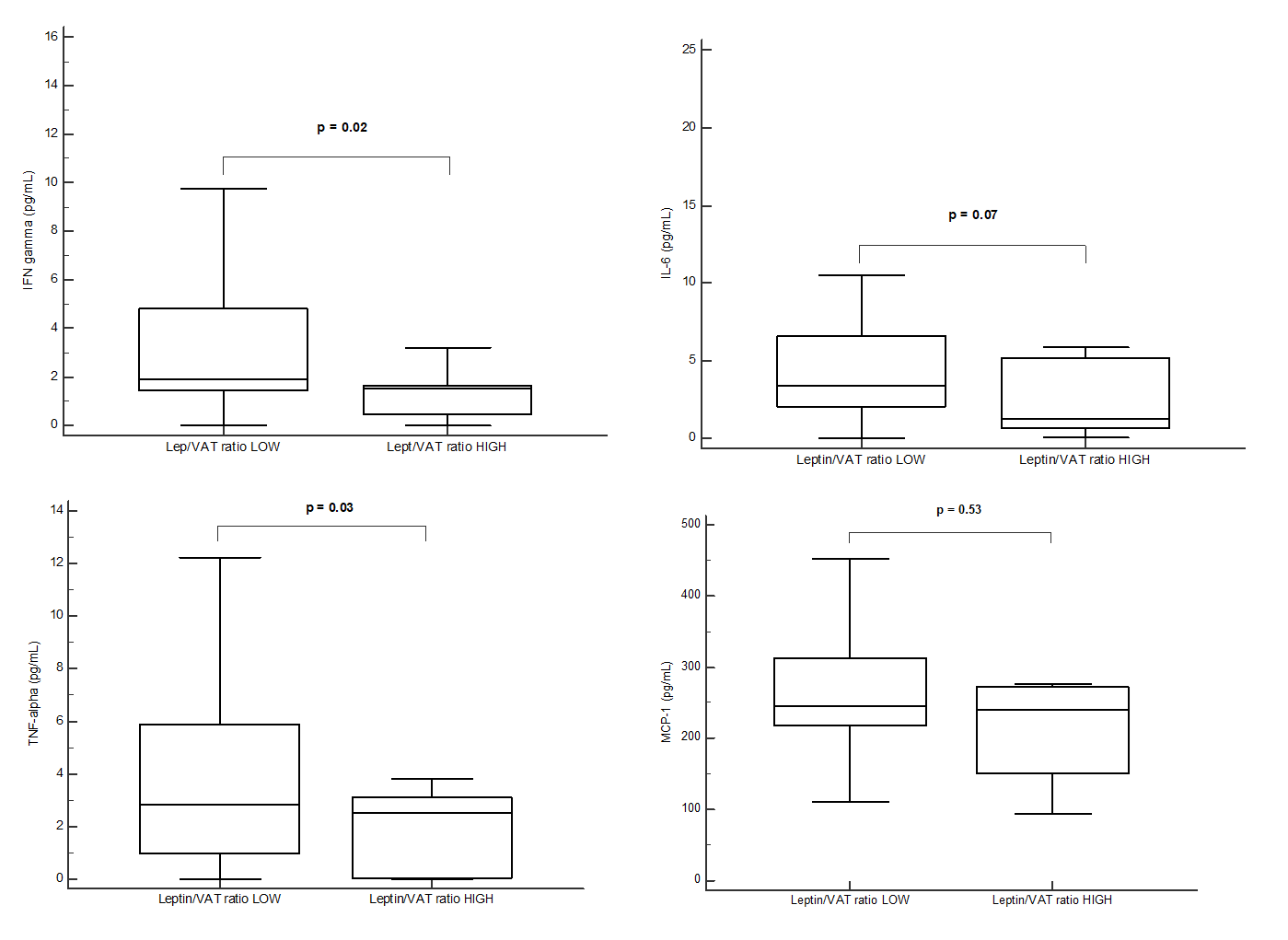
**Fig. S2. Boxplot of median cytokine blood concentration according to Leptin/VAT ratio subgroups**
